# Safety and health of professional drivers who drive on Brazilian highways

**DOI:** 10.1590/S1518-8787.2017051006761

**Published:** 2017-03-31

**Authors:** Fernanda Veruska Narciso, Marco Túlio de Mello

**Affiliations:** I Programa de Pós-Graduação em Ciências do Esporte. Universidade Federal de Minas Gerais. Belo Horizonte, MG, Brasil; II Departamento de Esportes. Universidade Federal de Minas Gerais. Belo Horizonte, MG, Brasil

**Keywords:** Transportation, manpower, Working Conditions, Occupational Risks, Shift Work, Accidents, Traffic, Occupational Health, Legislation, Labor

## Abstract

Traffic accidents and resulting injuries and deaths have become a global epidemic. In Brazil, most professional drivers, especially truck drivers, face irregular working hours and can be awake for more than 18 hours/day, which reduces their performance and alertness. In this article, we discuss the laws related to Brazilian professional drivers and their current amendments (No. 12,619/2012 and No. 13,103/2015) in relation to working hours at the wheel and rest breaks, which are vital for the quality of life of drivers and society in general. We note that the new law appears to be less efficient than the previous one as it causes insecurity and concern to the users of the transportation system, drivers, and employers. To restrict and reduce accidents, deaths, and injuries in traffic, appropriate legislation is essential, aiming at the safety of workers and users of highways. The law must also benefit the commercial aspect, strengthening the reduction in production and logistics losses. Additionally, traffic education programs are needed, as well as better supervision in relation to total working hours.

## RESUMO

Acidentes de trânsito com consequentes lesões e mortes têm se tornado uma epidemia em nível mundial. No Brasil, a maioria dos motoristas profissionais, sobretudo motoristas de transporte de cargas, enfrenta jornada de trabalho irregular e permanece acordado por mais de 18 horas/dia, o que reduz seu desempenho e estado de alerta. Neste artigo, discutimos as leis dos motoristas profissionais brasileiros e suas alterações vigentes (nº 12.619/2012 e nº 13.103/2015) em relação às horas de trabalho ao volante e a pausas para descanso, imprescindíveis para a qualidade de vida dos motoristas e para a sociedade em geral. Observamos que a nova legislação se mostra menos eficiente que a anterior por causar insegurança e preocupação aos usuários do sistema de transporte, aos próprios motoristas e aos empregadores. Para restringir e reduzir acidentes, mortes e lesões no trânsito, é fundamental uma legislação adequada, que vise à segurança do trabalhador e dos usuários das rodovias. A legislação deve, também, beneficiar o aspecto comercial, que se fortalece pela redução das perdas de produção e logística. Adicionalmente, são necessários programas de educação no trânsito e melhor fiscalização em relação ao tempo total de jornada de trabalho.

## INTRODUCTION

Injuries and deaths from accidents on highways have become a global epidemic, especially in developing countries, such as Brazil. In the United States, the rate of occurrence of fatal accidents caused by sleepy drivers was 21% between 2009 and and 2013^a^. In Brazil, the Federal Highway Police verified 168,593 accidents on Brazilian highways, amounting to 100,396 wounded and 8,227 dead persons in 2014. The lack of attention and sleepiness at the wheel caused 32.3% and 6% of the fatal accidents^b^, respectively. Given these alarming data, it becomes important to know and understand the causes or risk factors related to these accidents. The purpose of this article is to discuss the amendment of the Law related to professional drivers, No. 12,619 of Apr 30, 2012^c^, by Law No. 13,103 of Mar 2, 2015^d^ and the aspects related to sleep, working hours, and safety of drivers who drive on Brazilian highways.

### Sleepiness and Fatigue at the Wheel

Studies report several factors related to traffic accidents: alcohol intake when driving16; consumption of medicines and illicit drugs16,19; chronic sleep deprivation or restriction and sleep disorders5; lack of attention14; excessive sleepiness8,20; excessive working hours, monotony, and fatigue21,22; among others. Statistic data on traffic accidents from fatigue, sleepiness, and excessive working hours have worried researchers and various professionals in the world4,5,9,13,a. Recently, a study carried out with nineteen European countries13 has found that approximately 17% of the accidents were caused by falling asleep at the wheel. Of this percentage, the main causes were: poor sleep the night before (42.5%) and bad sleeping habits (34.1%). Nevertheless, one of the decisive reasons for falling asleep at the wheel was excessive sleepiness13.

In global terms, approximately 7% to 30% of fatal traffic deaths are a result of sleepiness and fatigue13,a. In the case of Brazilian drivers especially those who work irregular shifts, tiredness, fatigue, excessive working hours, lack of sleep, and short time of rest are a result of the excessive workload to meet deadlines and delivery times of goods (cargo) contained in their vehicles. Nascimento et al.16 have shown that 69% of Brazilian truck drivers drive ≥ 9 hours/day, 32% rest or sleep less than four hours, 66% use amphetamines while driving, and 91% drink alcohol while working. The reasons reported by the drivers for drinking alcohol were: social participation among friends, escape from routine, anxiety, and problems. The reasons for the use of amphetamine were: being in a hurry to go home, greater number of trips, and pressure from the company. A moderate correlation between the few hours of rest or sleep and the involvement in accidents under the influence of alcohol has also been observed by the authors. Sinagawa et al.19 have proved amphetamine-positive urine among truck drivers (9.9%) who drive long distances (> 270 km), of which 96% (n = 203) justify the use by the need to stay awake. Thus, the authors have concluded that these drivers use illegal drugs to combat fatigue during long distance journeys.

Another study has identified that 68.6% of truck drivers drive for more than 10 hours without a rest break and short sleep (between 5 and 6 hours), 43.8% ingest alcohol, 18.3% snore, and 26.5% report prior accident caused by sleepiness at the wheel5. Comparing Portuguese and Brazilian truck drivers, researchers21 have found that Brazilian drivers drive longer (> 16 hours) and have worse rates of sleepiness and quality of life in relation to general and mental health. The drivers of both countries have high prevalence of sleep disorders, high consumption of alcohol and psychostimulants, and high rate of accidents in the last five years20.

Given the above, we can see the need to implement more incisive public policies in relation to the health and safety of these workers, who drive through the highways of the country. Discussions about current laws in relation to working hours at the wheel, shift work, rest breaks, and restorative sleep are essential to the quality of life of drivers and society in general which uses the Brazilian road system.

### Working hours of Truck Drivers

Worldwide, most truck drivers face irregular working hours or shift work and stay awake for more than 18 hours/day. Studies indicate that by being awake for more than 19 hours the psychomotor performance is reduced, which is equivalent to high amounts of alcohol in the blood^2^ ^,^ ^8^. An example of irregular shift workers is the self-employed truck driver who works more than 12/14 hours on irregular schedules and various shifts, with a few hours of sleep (4 to 6 hours). Drivers shift workers change their work schedules according to a scale set by the company. In these two cases, most of the time, the sleep-wake cycle is inverted^15^, which harms the quality of sleep, making it inadequate and with repeated awakenings^18^.

### Laws established by some countries of America, Europe, and Australia

Researchers have developed studies on the working hours of professional drivers in many parts of the world. In general, countries apply laws or regulations in order to settle the tiredness and fatigue of these workers, enforcing rest intervals and limiting the hours at the wheel. Table 1 describes the general aspects of the labor laws related to truck drivers in the European Union and in countries such as United States, Australia, and Canada.

**Table 1:** International labor laws related to professional truck drivers.

International labor laws related to professional truck drivers	United States	European Union	Australia	Canada
Rest/24 hours	10 hours	11 hours	Minimum of 7 hours	10 hours
Limit of hours with rest break	8 hours with rest break	4 hours and 30 minutes: can be fractionated (30 min + 15 min)	5 hours	8 hours with rest break
Rest breaks	30 minutes	45 minutes	15 minutes	2 minutes
Daily working hours	11 hours	9 hours	12 hours	13 hours

Source: Goel^10^ (2012); Goel and Rousseau^12^ (2012); Goel et al.^11^ (2012).

In Brazil, the labor laws related to the driving and rest time of professional drivers have suffered changes over three years (Table 2). Driving time increased from 4 hours to 5h30 (Laws No. 12,619/2012 and No. 13,103/2015, respectively). Can one more hour and a half without rest break impair the attention and vigilance of drivers? Recent studies have found a high prevalence of sleepiness at the wheel and accidents with drivers who short sleep and drive for many hours without rest^8^ ^,^ ^17^. Research with drivers from Colombia and New Zealand have shown that excessive working hours (> 12 hours) at the wheel and sleeping less than 6 hours are risk factors for fatigue and accidents^9^ ^,^ ^22^.

**Table 2:** Old (12,619/2012) and new (13,103/2015) labor Laws related to professional truck drivers in Brazil.

Articles of Brazilian labor Laws related to professional truck drivers	Old Law (12,619/2012)	New Law (13,103/2015)
Rest/24 hours	11 horas: podendo ser fracionadas (9h + 2h)	11 hours: can be fractioned, enjoyed on the vehicle and coincide with the 30 minute intervals
Limit of uninterrupted hours (without rest break)/24 hours	4 hours	5 hours and 30 minutes
Rest breaks/24 hour	30 minutes: can be fractionated	30 minutes: can be fractionated
Daily working hours/24 hours	8 hours or by collective agreement: extension of up to 2 hours	8 hours: extension of up to 4 hours (by collective agreement or convention)
Driving of the vehicle in pairs of drivers (rotation): Rest	Rest of, at least, 6 consecutive hours outside of the vehicle or in the cabin bed with vehicle parked	Rest with moving vehicle, as long as there is a rest break every 72 hours of, at least, 6 hours outside of the vehicle or in cabin bed with vehicle parked

Source: Brasil. Lei nº 12.619, de 30 de abril de 2012. Dispõe sobre o exercício da profissão de motorista; altera a Consolidação das Leis do Trabalho - CLT, aprovada pelo Decreto-Lei nº 5.452, de 1º de maio de 1943 [...] e dá outras providências. *Diario Oficial da Uniao* Brasília, DF, 30 abr 2012 [cited 2017 Jan 20]. Available from: https://www.planalto.gov.br/ccivil_03/_ato2011-2014/2012/lei/l12619.htm and Brasil. Lei nº 13.103, de 2 de março de 2015. Dispõe sobre o exercício da profissão de motorista; altera a Consolidação das Leis do Trabalho - CLT, aprovada pelo Decreto-Lei nº 5.452, de 1º de maio de 1943 [...] e dá outras providências. *Diario Oficial da Uniao* Brasília, DF, 2 mar 2015 [cited 2017 Jan 20]. Available from: http://www.planalto.gov.br/ccivil_03/_Ato2015-2018/2015/Lei/L13103.htm

Regarding the rotation of drivers during the trip, the old law assured the rest of the drivers with the vehicle parked. The new law, however, has designated that drivers can sleep with the vehicle in motion. How can they obtain a restorative sleep in a moving vehicle? The National Sleep Foundation^e^ recommends sleeping according to the biological need, without fragmenting it, and in an environment free from disruptions. In this way, we can observe that the law disregards these actions regarding the aspect of sleep quality, sleep efficiency, and sleep hygiene during the rest period.

With this, we can note that the changes regulated by Law No. 13,103/2015 contribute to longer working hours and reduced rest time, harming the restorative sleep, attention, and alertness of drivers, which can cause sleepiness and accidents.

Given this scenario, it is undeniable that long working hours and cumulative sleep debt may cause changes in the biological rhythms and reduce the psychomotor performance, as well as cause accidents^1^ ^,^ ^6^. The Figure shows some factors that can trigger an accident.

**Figure:**
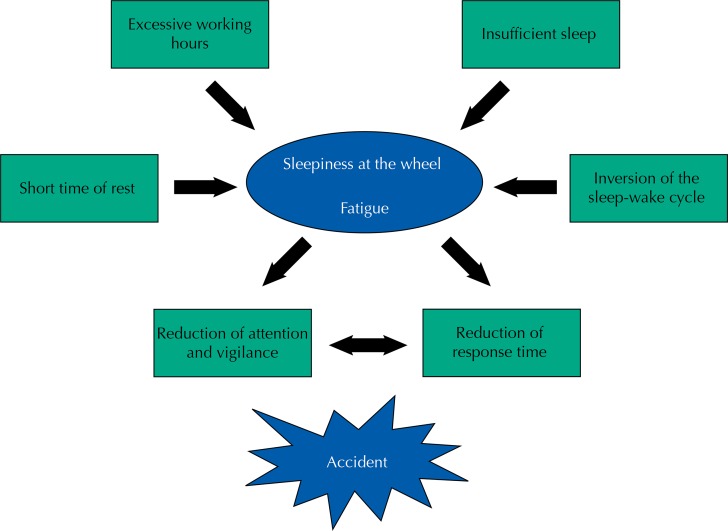
Factors that can trigger an accident.

Another resolution established in 2008 and adjusted in 2012 by the Brazilian National Transit Council (CONTRAN/DENATRAN – Resolutions No. 267 of Feb 15, 2008 and No. 425 of Nov 27, 2012^f^) to reduce the number of accidents and deaths caused by sleepy and fatigued drivers^3^, although relevant to the Brazilian society, has not been required in practice during evaluations for renewal, addition, and changes for the Brazilian driving license categories C, D, and E, and it has been much less enforced by transit agencies. Thus, this hinders the control and reduction of risks of accidents in Brazilian highways which are a result from sleep deprivation and disorders. We know that sleepiness and fatigue caused by sleep disorders, sleep deprivation, or sleep restriction directly affect the health and safety of drivers^4^ ^,^ ^15^. In this way, it is essential the compliance with the clinical investigations related to that resolution, in addition to a stricter supervision.

Regarding the health and safety of drivers, we used in this study the Fatigue Risk Index (FRI) (software Quineti Q for HSE)^7^ simulated in a period of 30 days, with three working hours (8, 10, and 12 hours), day and night shifts, 11 hours of rest time/24 hours, 48 hours off, limit of hours at the wheel (4 hours and 5h30), and rest breaks of 30 minutes, being them compatible to the two laws of 2012 and 2015. According to the authors^7^, the FRI is related to the likelihood of high levels of sleepiness, and the result is expressed by a value between zero and 100. An index of 20.7 is considered an average value of fatigue for a working schedule of two days of day shift, two days of night shift, and four days off. The average relative risk of an accident or incident (RRA/I) is equal to one (1.0). The results for the FRI and RRA/I are shown in Table 3.

**Table 3:** Average values for the FRI and RRA/I for 8, 10 and 12 working hours during 30 days (simulation).

Working hours	Limit of hours at the wheel	Average FRI	Average RRA/I
8 hours	4 hours	15,4	1,05^b^
	5h30	18,8	1,09^b^
10 hours	4 hours	22,7^a^	1,44^b^
	5h30	27,1^a^	1,49^b^
12 hours	4 hours	32,5^a^	2,20^b^
	5h30	37,8^a^	2,27^b^

FRI: fatigue/risk index; RRA/I: average relative risk of an accident or incident

^a^ Values for the FRI above average (20.7).

^b^ Values for the RRA/I above average (1.0)^7^.

The results show increased fatigue and risk of accident every two more working hours and every 1h30 more of driving without a rest break. It is important to mention that the risk of an accident doubles after 12 working hours and increases when working 5h30 (RRA/I = 2.27) compared to 4 hours (RRA/I = 2.20). This has also been noted in prior studies that have associated risks of accidents when driving over long distances and working ≥ 2 consecutive hours^6^ ^,^ ^17^. In this way, it is relevant to point out that these 2 and 4 more hours driving (10 and 12 hours) are provided for in the laws mentioned above as overtime work and they are not consistent with the improvement of road safety, the work at the wheel, and, much less, the safety of the population.

We suggest that the new law related to drivers appears to be less efficient than the previous one as it causes insecurity and concern to the users of the transportation system, drivers, and employers. Unfortunately, we realize that the Brazilian authorities and society still have no knowledge of the exponential increase of the risk of accidents concerning the lack of sleep and the excess of wakefulness that these drivers are induced to practice. In this sense, we can observe the complacency of employers and the disregard of the Brazilian legislation, as well as the lack of commitment of the drivers themselves to the prudence at the wheel and to the users of the road network.

## FINAL CONSIDERATIONS

We emphasize the urgent need for specific actions in relation to traffic accidents and deaths caused mainly by overwork and insufficient sleep. We can propose some appropriate and relevant aspects to be implemented: 1) reduced driving time; 2) more rest breaks during working hours; 3) scheduled naps; 4) more days off on weekends; 5) leisure activities and practice of physical activity; 6) quality and longer night sleep; and, 7) drivers should avoid working or staying awake during the dark phase (night shift) in order to maintain the synchronization of biological rhythms. Through educational and awareness actions aimed at drivers and employers, these key strategies become essential to improve the lifestyle of drivers and to provide a safer driving, in order to resolve or eradicate this major public health problem: the high number of traffic accidents and deaths resulting from sleepiness and fatigue.

## Footnotes

a Teft BC. Prevalence of motor vehicle crashes involving drowsy drivers, United States, 2009-2013. Washington (DC): American Automobile Association Foundation for Traffic Safety; 2014 [cited 2017 Jan 20]. p.1-8. Available from: https://www.aaafoundation.org/sites/default/files/AAAFoundation-DrowsyDriving-Nov2014.pdf

b Ministério da Justiça (BR), Departamento de Polícia Rodoviária Federal. Prestação de contas ordinária anual. Relatório de gestão do exercício de 2014. Brasília (DF): DPRF/MJ; 2015 [cited 2017 Jan 20]. Available from: https://www.prf.gov.br/portal/estados/sede/prestacao-de-contas/2014-relatorio-de-gestao

c Brasil. Lei nº 12.619, de 30 de abril de 2012. Dispõe sobre o exercício da profissão de motorista; altera a Consolidação das Leis do Trabalho - CLT, aprovada pelo Decreto-Lei no 5.452, de 1º de maio de 1943 [...] e dá outras providências. *Diario Oficial Uniao*. 30 abr 2012 [cited 2017 Jan 20]. Available from: https://www.planalto.gov.br/ccivil_03/_ato2011-2014/2012/lei/l12619.htm

d Brasil. Lei nº 13.103, de 2 de março de 2015. Dispõe sobre o exercício da profissão de motorista; altera a Consolidação das Leis do Trabalho - CLT, aprovada pelo Decreto-Lei no 5.452, de 1º de maio de 1943 [...] e dá outras providências. *Diario Oficial Uniao*. 2 mar 2015 [cited 2017 Jan 20]. Available from: http://www.planalto.gov.br/ccivil_03/_Ato2015-2018/2015/Lei/L13103.htm

e National Sleep Foundation. Sleep in American poll: summary of findings. Washington (DC): National Sleep Foundation; 2008.

f Conselho Nacional de Trânsito. Resolução nº 425, de 27 de novembro de 2012. Dispõe sobre o exame de aptidão física e mental, a avaliação psicológica e o credenciamento das entidades públicas e privadas de que tratam o art. 147, I e §§ 1º a 4º e o art. 148 do Código de Trânsito Brasileiro. *Diario Oficial da Uniao.* 27 nov 2012 [cited 2017 Jan 20]. Available from: https://www.legisweb.com.br/legislacao/?id=247963

## References

[1] Åkerstedt T, Wright KP (2009). Sleep Loss and Fatigue in Shift Work and Shift Work Disorder. Sleep Med Clin.

[2] Dawson D, Reid K (1997). Fatigue, alcohol and performance impairment. Nature.

[3] de Mello MT, Bittencourt LR, Cunha Rde C, Esteves AM, Tufik S (2009). Sleep and transit in Brazil: new legislation. J Clin Sleep Med.

[4] de Mello MT, Narciso FV, Tufik S, Paiva T, Spence DW, Bahammam AS (2013). Sleep disorders as a cause of motor vehicle collisions. Int J Prev Med.

[5] de Pinho RS, da Silva FP, Bastos JP, Maia WS, de Mello MT, de Bruin VM (2006). Hypersomnolence and accidents in truck drivers: A cross-sectional study. Chronobiol Int.

[6] Folkard S, Lombardi DA (2006). Modeling the impact of the components of long work hours on injuries and “accidents”. Am J Ind Med.

[7] Folkard S, Robertson KA, Spencer MB (2007). A Fatigue/Risk index to assess work schedules. Somnologie.

[8] Ftouni S, Sletten TL, Howard M, Anderson C, Lenne MG, Lockley SW (2013). Objective and subjective measures of sleepiness, and their associations with on-road driving events in shift workers. J Sleep Res.

[9] Gander PH, Marshall NS, James I, Le Quesne L (2006). Investigating driver fatigue in truck crashes: Trial of a systematic methodology. Transp Res Part F Traffic Psychol Behav.

[10] Goel A (2012). The minimum duration truck driver scheduling problem. EURO J Transp Logist.

[11] Goel A, Archetti C, Savelsbergh M (2012). Truck driver scheduling in Australia. Comput Oper Res.

[12] Goel A, Rousseau LM (2012). Truck driver scheduling in Canada. J Sched.

[13] Gonçalves M, Amici R, Lucas R, Akerstedt T, Cirignotta F, Horne J (2015). Sleepiness at the wheel across Europe: a survey of 19 countries. J Sleep Res.

[14] Ledesma RD, Montes SA, Poó FM, López-Ramón MF (2010). Individual differences in driver inattention: the attention-related driving errors scale. Traffic Inj Prev.

[15] Moreno CR, Louzada FM, Teixeira LR, Borges F, Lorenzi G (2006). Short sleep is associated with obesity among truck drivers. Chronobiol Int.

[16] ECd Nascimento, Nascimento E, JdP Silva (2007). Uso de álcool e anfetaminas entre caminhoneiros de estrada. Rev Saude Publica.

[17] Phillips RO, Sagberg F (2013). Road accidents caused by sleepy drivers: Update of a Norwegian survey. Accid Anal Prev.

[18] Santos EH, de Mello MT, Pradella-Hallinan M, Luchesi L, Pires ML, Tufik S (2004). Sleep and sleepiness among Brazilian shift-working bus drivers. Chronobiol Int.

[19] Sinagawa DM, De Carvalho HB, Andreuccetti G, Do Prado NV, De Oliveira KC, Yonamine M (2015). Association between travel length and drug use among Brazilian truck drivers. Traffic Inj Prev.

[20] Souza JC, Paiva T, Reimão R (2008). Sono, qualidade de vida e acidentes em caminhoneiros brasileiros e portugueses. Psicol Estud.

[21] Thiffault P, Bergeron J (2003). Monotony of road environment and driver fatigue: a simulator study. Accid Anal Prev.

[22] Torregroza-Vargas NM, Bocarejo JP, Ramos-Bonilla JP (2014). Fatigue and crashes: The case of freight transport in Colombia. Accid Anal Prev.

